# Supplementary material for: DNA methylation in newborns conceived by assisted reproductive technology
Source: Nat Commun. 2022 Apr 7;13:1896. doi: 10.1038/s41467-022-29540-w (PMC8989983; doi:10.1038/s41467-022-29540-w)
Supplement: Supplementary file 1 — Supplementary Information [file 41467_2022_29540_MOESM1_ESM.pdf]

Supplementary information

Supplement to Håberg SE, Page CM, Lee Y, et al. DNA Methylation In newborns conceived by assisted reproductive technology

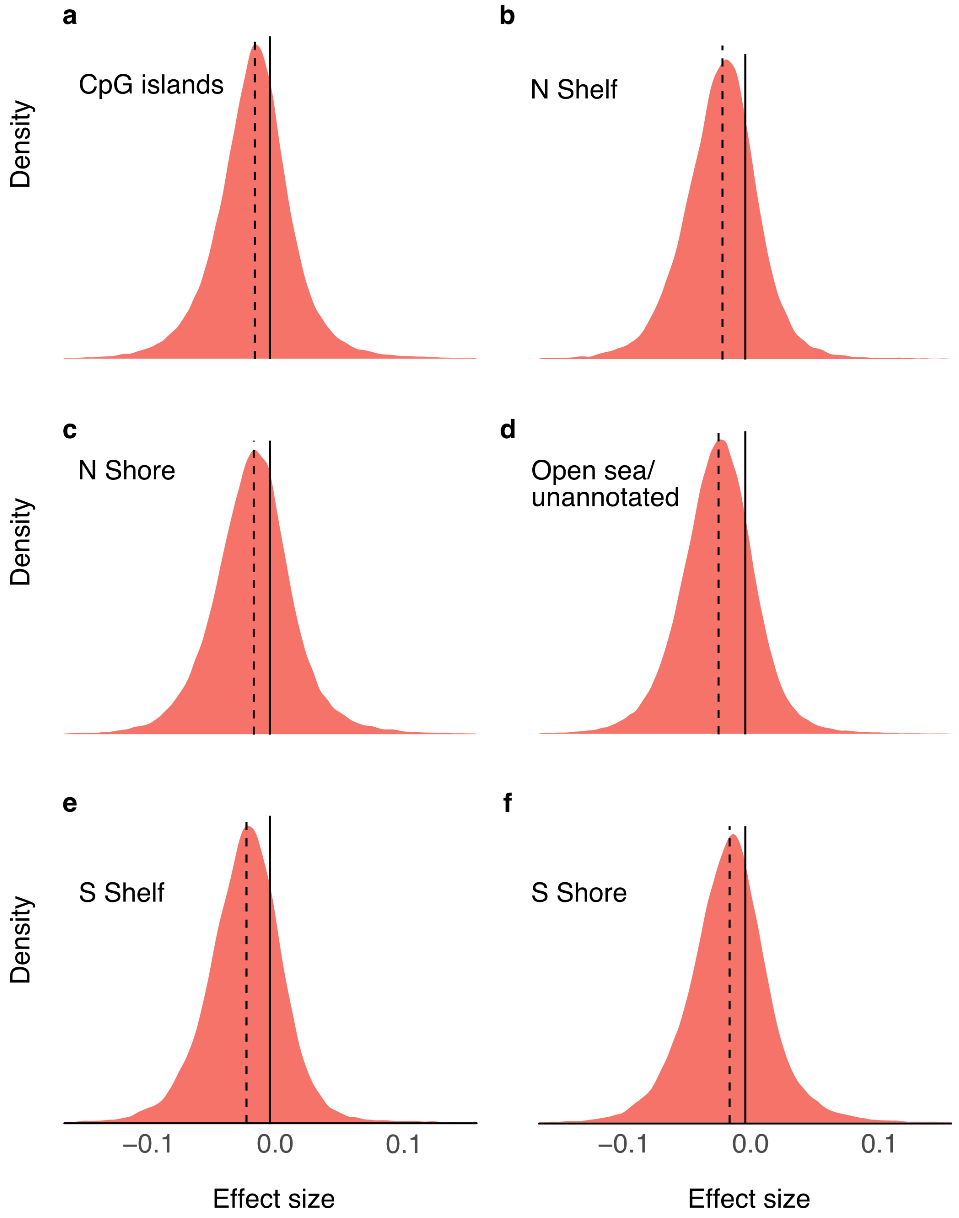

Supplement Figure 1 Distribution of the differences in DNA methylation between ART conceived and naturally conceived according to genomic feature.
The effect size on the x-axis refers to the coefficients from the regression of DNA methylation at each CpG site on ART conceived versus naturally conceived newborns. The density on the y-axis was scaled. The vertical dashed line indicates the median value of the effect sizes, and the vertical solid black line indicates zero. **a** CpG islands, **b** North shelf, **c** North shore, **d** Open sea/unannotated, **e** South shelf, **f** South shore. Source data are provided as a Source Data file

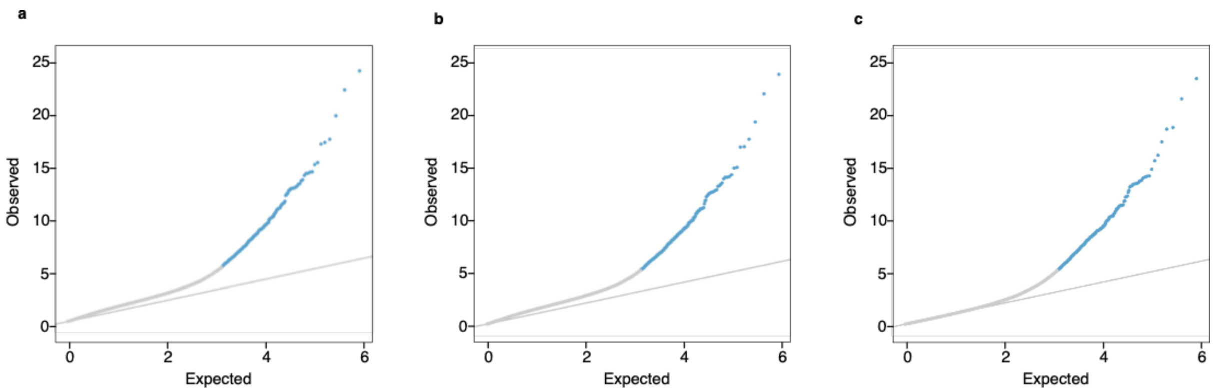

Supplement Figure 2 Quantile-Quantile plot of observed against expected -$\log_{10}$ *P* values for the differences in DNA methylation between ART and naturally conceived newborns, with different sets of adjustment variables.
a The observed –$\log_{10}$ *P* values (calculated from *t* statistics) were derived from two-sided *t* tests for comparing the DNA methylation levels between ART conceived newborns (n=962) and naturally conceived newborns (n=983) adjusted for maternal age, maternal smoking, maternal BMI, child sex, parity, and plate ID (the main model).
b Adjusted for the covariates in **a** and gestational age at birth.
c Adjusted for the covariates in **a** and gestational age at birth, birthweight, maternale ducation level, paternal age, and cord blood cell composition.
Blue dots are the CpGs at FDR <0.01, while grey dots denote nonsignificant CpGs.
Source data are provided as a Source Data file.

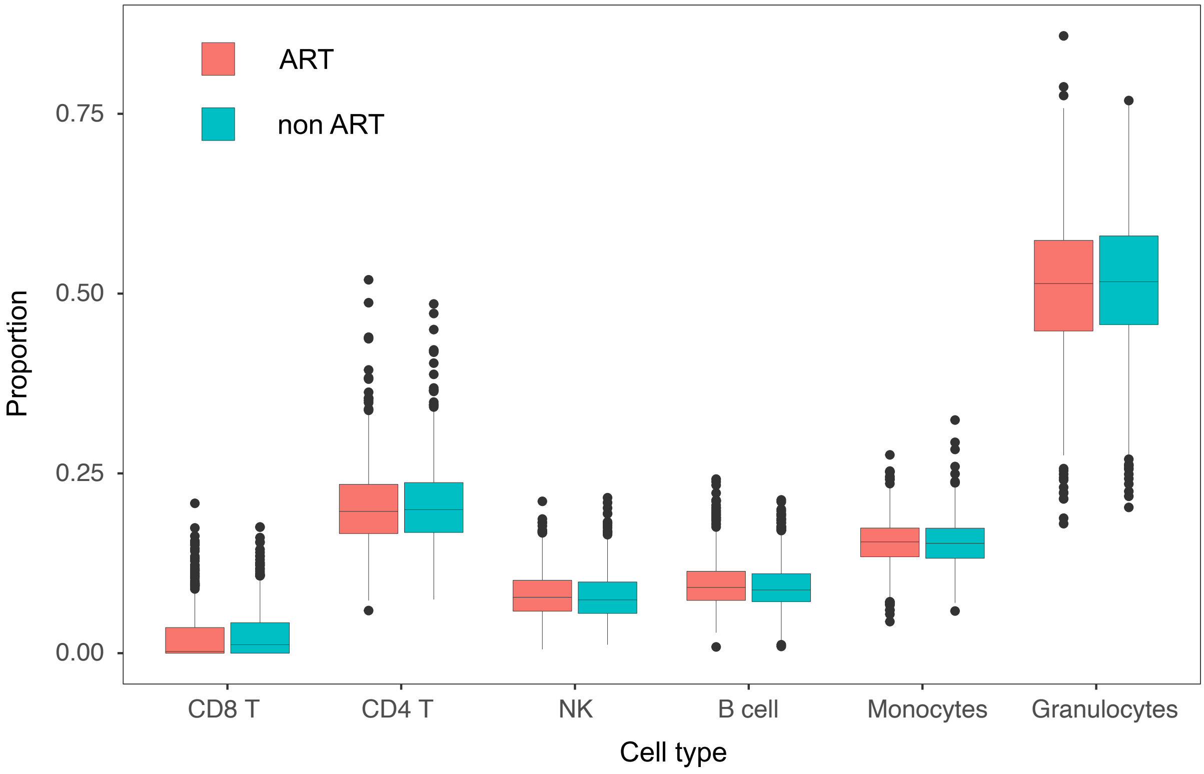

Supplement Figure 3 Differences in the cord blood cell composition between ART conceived and naturally conceived newborns.
The upper and lower box limits correspond to the interquartile range (25% to 75% of the values for each cell type). The horizontal center line in the boxes represents the median values, and the vertical lines represent 1.5 times the interquartile range. The black points represent values outside this range**.** In this figure, 962 ART conceived and 983 naturally conceived newborns were included. Source data are provided as a Source Data file.

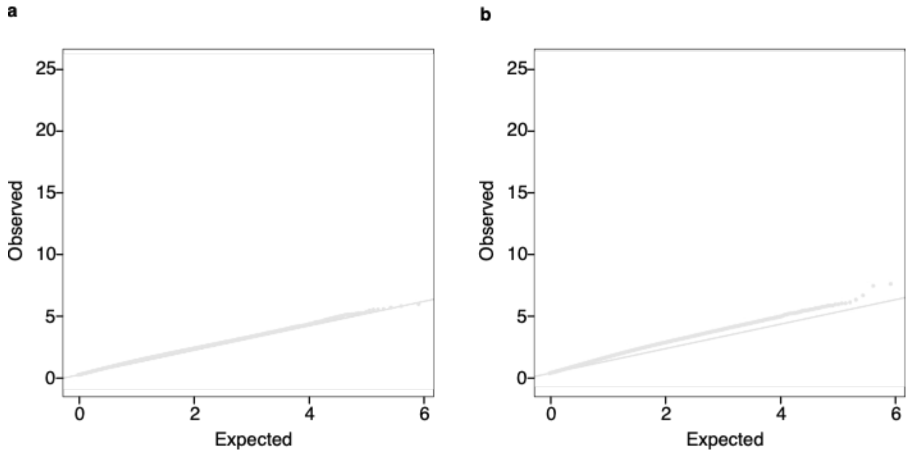

Supplement Figure 4 Quantile-Quantile plots of expected versus observed -$\log_{10}$ *P* values for the differences in DNA methylation with increasing time to pregnancy.

a The observed –log10 P values (calculated from t statistics) were derived from two-sided t tests for associating the DNA methylation levels and the time to pregnancy (TTP), with adjustment for maternal age, maternal smoking, maternal BMI before pregnancy, child sex, parity, and plate ID.

b The observed –log10 P values (calculated from t statistics) were derived from two-sided t tests for the differences in DNA methylation between TTP >12 months and TTP <3 months. The same adjusting variables were used. Grey dots refer to insignificant CpGs.

Source data are provided as a Source Data file.

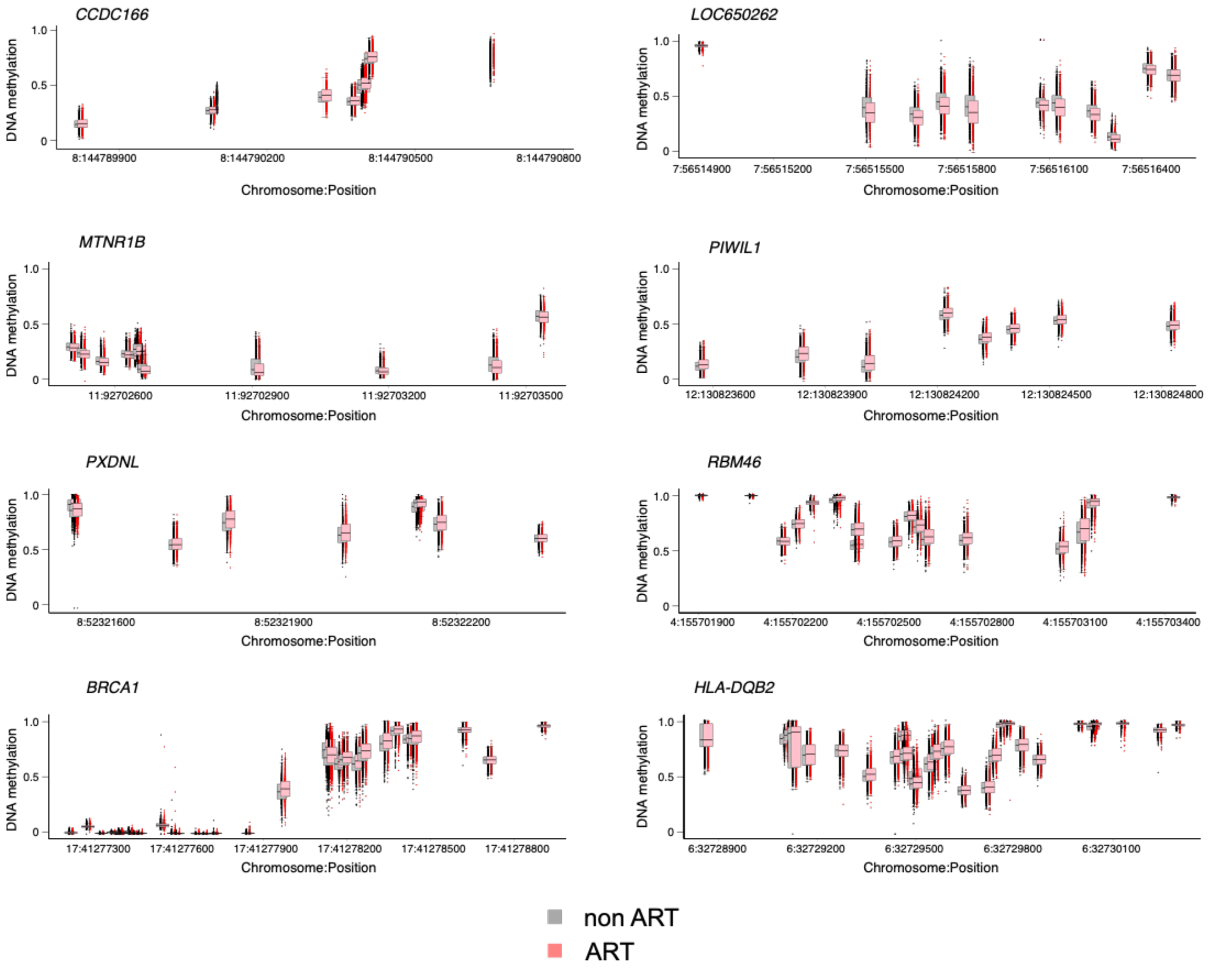

Supplement Figure 5 Distribution of the methylation level at differentially methylated regions for the eight genes with more than eight significantly differentially methylated CpGs between ART and naturally conceived newborns.

The DNA methylation levels between 962 ART conceived and 983 naturally conceived newborns were examined. The box shows the interquartile range (from the 25th to 75th percentile), and the middle line in the box represents the median value. The whiskers outstretch 1.5 times the box height from the top and bottom of the box. If the whiskers reach beyond the minimum and maximum value, they are truncated at the minimum and maximum value. The dots outside the whiskers represent outliers beyond the interquartile range. Source data are provided as a Source Data file.

Supplement Table 1 Overlap of differentially methylated CpGs in newborns conceived wi th frozen and fresh embryo transfer.

Gene [a]	chr:pos [b]	CpG probe name	Differentially methylated CpGs FDR<0.01 in both comparisons		Differentially methylated CpGs FDR<0.01
			Frozen vs natural Effect size	Fresh vs natural Effect size	Frozen vs fresh Effect size
APC2	chr19:1465556	cg12154045	-0.265	-0.223	
APC2	chr19:1465962	cg22560193	-0.734	-0.559	
LOC644669	chr18:15321140	cg08958748	-0.272	-0.185	
KLC1	chr14:104153614	cg21496948			-0.224
	chr7:56551640	cg26733975			0.394
ASB6	chr9:132403257	cg00632853			0.252

[a] Official gene symbol
[b] Chromosome and Position on Chromosome
The effect sizes refer to the coefficients from the regressions of DNA methylation on a variable comparing two groups, e.g., frozen versus natural, fresh versus natural, or frozen versus fresh, with adjustment for maternal age, maternal smoking, maternal BMI, child sex, parity, and plate ID.

Supplement Table 2 Overlap of differentially methylated CpGs with other studies.

			Håberg et al.	Novakovic et al.[a]	Yeung et al.[b]
Gene [c]	chr:pos [d]	CpG probe name	Effect size [e]	Delta Methylation	Effect size
NECAB3	20:32254706	cg00478435	-0.192		
NECAB3	20:32255052	cg07470512	-0.572		
NECAB3	20:32255491	cg03904042	-0.471	-0.118	
NECAB3	20:32255988	cg14921437	-0.267		-0.026
NECAB3	20:32256071	cg13403462	-0.370		-0.030
CHRNE	17:4803684	cg22349396	-0.220		
CHRNE	17:4804104	cg06444025	-0.201		
CHRNE	17:4804357	cg01726265	-0.480		
CHRNE	17:4804674	cg10553748	-0.099	-0.064	
CHRNE	17:4804838	cg09495303	-0.172		
CHRNE	17:4805392	cg24768135	-0.208	-0.107	
GET1	21:40759534	cg19761502	-0.495		
GET1	21:40759555	cg04378774	-0.499		
GET1	21:40759574	cg27119318	-0.519		-0.049
GET1	21:40759659	cg21736411	-0.414		
GET1	21:40759686	cg19684207	-0.504		-0.048
GET1	21:40759694	cg15977137	-0.589		
KLK4	19:51411737	cg08681856	0.118		0.055
KLK4	19:51411782	cg03473127	0.107		
KLK4	19:51411839	cg11976052	0.089		
KLK4	19:51411940	cg27128734	0.167		
MYO1D	17:31149877	cg01050010	-0.403		-0.045

[a] Reported DMRs identified by DMRcate in Novakovic B, et al.[1]
[b] Reported differentially methylated CpGs, FDR < 0.05. Yeung EH,et al.[2]
[c] Official gene symbol
[d] Chromosome and Position on Chromosome
[e] Effect sizes refer to the coefficients from the regressions of DNA methylation on ART conceived versus naturally conceived with adjustment for maternal age, maternal smoking, maternal BMI, child sex, parity, and plate ID

References:

1. Novakovic B, Lewis S, Halliday J, et al. Assisted reproductive technologies are associated with limited epigenetic variation at birth that largely resolves by adulthood. Nat Commun. 2019 Sep 2;10(1):3922. doi: 10.1038/s41467-019-11929-9. PMID: 31477727; PMCID: PMC6718382.

2. Yeung EH, Mendola P, Sundaram R, et al. Conception by fertility treatment and offspring deoxyribonucleic acid methylation. Fertil Steril. 2021 Aug;116(2):493-504. doi: 10.1016/j.fertnstert.2021.03.011. Epub 2021 Apr 3. PMID: 33823999; PMCID: PMC8349775.
