# Supplementary material for: DNA methylation in newborns conceived by assisted reproductive technology
Source: Nat Commun. 2022 Apr 7;13:1896. doi: 10.1038/s41467-022-29540-w (PMC8989983; doi:10.1038/s41467-022-29540-w)
Supplement: Supplementary file 3 — Description of Additional Supplementary Files [file 41467_2022_29540_MOESM3_ESM.pdf]

Description of Additional Supplementary Files

File Name: Supplementary Data 1
Description: CpGs with significantly different DNA methylation (FDR < 0.01) between ART and naturally conceived newborns.

File Name: Supplementary Data 2
Description: Phenotypes associated with genes that have ≥ 2 differentially methylated CpGs.
