# Supplementary material for: DNA methylation in newborns conceived by assisted reproductive technology
Source: Nat Commun. 2022 Apr 7;13:1896. doi: 10.1038/s41467-022-29540-w (PMC8989983; doi:10.1038/s41467-022-29540-w)
Supplement: Supplementary file 6 — Reporting Summary [file 41467_2022_29540_MOESM6_ESM.pdf]

nature research

Corresponding author(s): Siri E. Håberg

Last updated by author(s): Jan 19, 2022

Reporting Summary

Nature Research wishes to improve the reproducibility of the work that we publish. This form provides structure for consistency and transparency in reporting. For further information on Nature Research policies, see Authors & Referees and the Editorial Policy Checklist .

Statistics

For all statistical analyses, confirm that the following items are present in the figure legend, table legend, main text, or Methods section.

n/a	Confirmed	
☐	☒	The exact sample size (*n*) for each experimental group/condition, given as a discrete number and unit of measurement
☐	☒	A statement on whether measurements were taken from distinct samples or whether the same sample was measured repeatedly
☐	☒	The statistical test(s) used AND whether they are one- or two-sided<br>*Only common tests should be described solely by name; describe more complex techniques in the Methods section.*
☐	☒	A description of all covariates tested
☐	☒	A description of any assumptions or corrections, such as tests of normality and adjustment for multiple comparisons
☐	☒	A full description of the statistical parameters including central tendency (e.g. means) or other basic estimates (e.g. regression coefficient) AND variation (e.g. standard deviation) or associated estimates of uncertainty (e.g. confidence intervals)
☐	☒	For null hypothesis testing, the test statistic (e.g. *F*, *t*, *r*) with confidence intervals, effect sizes, degrees of freedom and *P* value noted<br>*Give P values as exact values whenever suitable.*
☒	☐	For Bayesian analysis, information on the choice of priors and Markov chain Monte Carlo settings
☐	☒	For hierarchical and complex designs, identification of the appropriate level for tests and full reporting of outcomes
☐	☒	Estimates of effect sizes (e.g. Cohen's *d*, Pearson's *r*), indicating how they were calculated

Our web collection on statistics for biologists contains articles on many of the points above.

Software and code

Policy information about availability of computer code

Data collection	Raw data for the Illumina MethylationEPIC array was obtained using Genome Studio 2011.2
Data analysis	Methods and software used are stated in the methods section of the manuscript. We used: STATA (version 16) R (version: www.r-project.org; v3.5.0 and v3.5.2). Quality control (R 3.5.2): RnBeads R package (version 2.2.0), wateRmelon R package (version 1.26.0), ENmix.oob (within RnBeads) normalization (BMIQ) (within wateRmelon), Regression analysis (R 3.5.0): Rfast (version 1.9.2).

For manuscripts utilizing custom algorithms or software that are central to the research but not yet described in published literature, software must be made available to editors/reviewers. We strongly encourage code deposition in a community repository (e.g. GitHub). See the Nature Research guidelines for submitting code & software for further information.

Data

Policy information about availability of data

All manuscripts must include a data availability statement. This statement should provide the following information, where applicable:

- Accession codes, unique identifiers, or web links for publicly available datasets
- A list of figures that have associated raw data
- A description of any restrictions on data availability

Data availability
CpGs were annotated to include further information. The initial annotation was obtained from the Illumina EPIC manifest file (https://support.illumina.com/array/array_kits/infinium-methylationepic-beadchip-kit/downloads.html), which provides information on CpG probe ID, probe sequence, chromosome position, gene name etc. Approved gene names were obtained from HUGO Gene Nomenclature Committee (HGNC; https://www.genenames.org). In order to provide stable gene IDs, ENSEMBL IDs (www.ensembl.org) are included. Information on human genetic disorders was obtained from Online Mendelian Inheritance in Man (OMIM; https://www.omim.org). Information on mouse mutation phenotypes was obtained from Mouse Genome Informatics (MGI; http://www.informatics.jax.org).
The data that support the findings of this study are available from the Norwegian Institute of Public Health (NIPH), but restrictions apply regarding the availability of these data, which were originally used under specific approvals for the current study and are therefore not publicly available. The individual level are available under

restricted access due to regulations and can only be given after approval by the Norwegian Ethical committees approving that the applications are consistent with the consent provided. Access can be obtained by application to the Norwegian Institute of Public Health using a form available on the English language portion of their website at https://www.fhi.no/en/studies/moba /. Specific questions regarding access for data in this study can be directed to Siri.Haberg@fhi.no. The data generated in this study are provided in the Supplementary Information. Source data are provided with this paper. R scripts are available from the authors upon request.

Field-specific reporting

Please select the one below that is the best fit for your research. If you are not sure, read the appropriate sections before making your selection.

- [x] Life sciences
- [ ] Behavioural & social sciences
- [ ] Ecological, evolutionary & environmental sciences

For a reference copy of the document with all sections, see nature.com/documents/nr-reporting-summary-flat.pdf

Life sciences study design

All studies must disclose on these points even when the disclosure is negative.

Sample size	This study included a subset of the MoBa mother-father-child trios who met the following criteria: the children were singletons born 2001-20099 with a record in the MBRN, mothers had returned the first MoBa questionnaire around pregnancy week 18, and DNA samples from the complete trios (mother-father-child) were available. The sample selection is detailed in Fig. 1. Among the trios who met the inclusion criteria, we randomly selected 992 trios with non-ART conceived children and 978 trios with ART conceived children.
Data exclusions	During quality control, we excluded two children with empty plate wells, one child with outlier values, three children with corrupt images, and 19 children with high background signals, leaving 1945 children and their parents for the current analyses. After receiving the iDAT files, we performed quality control using the RnBeads R package in four separate batches. We removed 44,210 cross-hybridizing probes and 16,117 additional probes where the last three bases overlapped with a SNP. Probes with a high detection p value (> 0.01) were also removed. In addition, the different batches were subjected to the greedycut algorithm to remove samples and probes with outlying DNA methylation patterns. The remaining DNA methylation signals were corrected for background noise using the normalizing function enmix.oob. The signal intensity of all the samples was visually inspected using the output of control probes from RnBeads. Whenever a CpG site was removed from one batch due to poor quality or high detection p value, it was subsequently removed from all remaining batches. This resulted in 770,586 autosomal CpGs in the final set for the current analyses.
Replication	We did not attempt to replicate our findings in an independent cohort. To our knowledge, there are no other cohorts available with sufficient sample size on ART to perform the different analyses on subgroups in line with our analyses. We found no other cohort with similar data on mother-father-child trios with DNA methylation in ART and non-ART trios.
Randomization	Study participants who fulfilled criteria were randomly selected. To account for differences between cases and controls, we adjusted for potential confounders. Parents who use ART to conceive are known to differ from those who conceive naturally in several ways. Parental and other characteristics of ART conceived and non-ART conceived children are listed in Table 1. All samples were randomly placed on plates before measuring DNA methylation to reduce batch effects
Blinding	The study was based on an ongoing cohort study with participants enrolled during pregnancy around the week 18 of pregnancy. Assisted conceptions was the exposure of interest and registered in birth records at delivery. Blinding of conception method was not an option.

Reporting for specific materials, systems and methods

We require information from authors about some types of materials, experimental systems and methods used in many studies. Here, indicate whether each material, system or method listed is relevant to your study. If you are not sure if a list item applies to your research, read the appropriate section before selecting a response.

Materials & experimental systems

n/a	Involved in the study	
x		Antibodies
x		Eukaryotic cell lines
x		Palaeontology
x		Animals and other organisms
	x	Human research participants
x		Clinical data

Methods

n/a	Involved in the study	
x		ChIP-seq
x		Flow cytometry
x		MRI-based neuroimaging

Human research participants

Policy information about studies involving human research participants

Population characteristics	Parental and other characteristics of ART conceived and non-ART conceived children are listed in Table 1.
Recruitment	The study was based on a subsample of particpiants in the large Norwegian Mother, Father and Child cohort study, in which more than 95 000 pregnant women and partners were recruited during around 15-18 weeks of gestation between 1999 and 2008. Parents who use ART to conceive differ from those who conceive without ART in several ways. We were able to control for potential confounders that have been associated with both the use of ART and with DNA methylation in newborns, such as maternal age, maternal smoking, and maternal BMI. However, as information on some of these potential confounders and other key variables was derived from questionnaires, potential misclassification may have limited our ability to fully account for confounders. Time to pregnancy (TTP) is difficult to capture precisely, although long and short TTPs are more reliably recalled. Misclassifications may weaken an association and reduce the ability to detect differences with TTP. Although women included in MoBa are not completely representative of all Norwegian mothers, the biological implications are expected to be similar for all women and children. This is supported by a study showing that associations between pregnancy exposures and child outcomes were not biased by selection into the MoBa study.
Ethics oversight	This study was approved by the institutional review board at the Norwegian Institute of Public Health and by the Regional Ethics committee of South East Norway (#2017/1362). The establishment of MoBa and initial data collection was based on a license from the Norwegian Data Protection Agency and approval from the Regional Committees for Medical and Health Research Ethics. The MoBa cohort is now based on regulations related to the Norwegian Health Registry Act. All participants provided written informed consent. There were no participant compensation.More information is availbale here: https://www.fhi.no/en/studies/moba/for-forskere-artikler/questionnaires-from-moba/ .

Note that full information on the approval of the study protocol must also be provided in the manuscript.
